# Effect of Supplementation of a Butyrate-Based Formula in Individuals with Liver Steatosis and Metabolic Syndrome: A Randomized Double-Blind Placebo-Controlled Clinical Trial

**DOI:** 10.3390/nu16152454

**Published:** 2024-07-28

**Authors:** Federica Fogacci, Marina Giovannini, Valentina Di Micoli, Elisa Grandi, Claudio Borghi, Arrigo Francesco Giuseppe Cicero

**Affiliations:** Hypertension and Cardiovascular Risk Research Unit, Medical and Surgery Sciences Department, Alma Mater Studiorum University of Bologna, 40138 Bologna, Italy; marina.giovannini3@unibo.it (M.G.); valentina.dimicoli@gmail.com (V.D.M.); elisa.grandi@unibo.it (E.G.); claudio.borghi@unibo.it (C.B.); arrigo.cicero@unibo.it (A.F.G.C.)

**Keywords:** butyrate, clinical trial, dietary supplement, NAFLD, postbiotics

## Abstract

Postbiotics could exert different metabolic activities in animal models of non-alcoholic fatty liver disease (NAFLD) and in humans affected by metabolic syndrome. This is a randomized, double-blind, placebo-controlled, parallel-group clinical trial that enrolled a sample of 50 Caucasian healthy individuals with NAFLD, defined as liver steatosis, and metabolic syndrome. After a 4-week run-in, the enrolled individuals were randomized to take a food for special medical purposes with functional release, one tablet a day, containing calcium butyrate (500 mg/tablet), zinc gluconate (zinc 5 mg/tablet), and vitamin D3 (500 IU/tablet), or an identical placebo for 3 months. Liver and metabolic parameters were measured at baseline and at the end of the study. No subject experienced any adverse events during the trial. In both groups, a significant decrease in total cholesterol (TC) and triglycerides (TG) plasma levels was observed at the randomization visit vs. pre-run-in visit (*p* < 0.05). Regarding liver parameters, after treatment, the fatty liver index (FLI) improved significantly vs. baseline values (*p* < 0.05) and vs. placebo group (*p* < 0.05) in the active treatment group, and the hepatic steatosis index (HSI) improved significantly vs. baseline values (*p* < 0.05). Moreover, after active treatment, TC, TG, and gamma-glutamyl transferase (gGT) improved significantly vs. baseline values (*p* < 0.05), and TC and TG improved vs. placebo group (*p* < 0.05), as well. In the placebo group, liver parameters remained unchanged after treatment; only TG improved significantly vs. baseline values (*p* < 0.05). In our study, we observed that the butyrate-based formula improved FLI and plasma lipid patterns in individuals affected by liver steatosis and metabolic syndrome.

## 1. Introduction

Non-alcoholic fatty liver disease (NAFLD) is emerging as the most common chronic liver disease globally. It is a well-known risk factor for cardiovascular disease (CVD) and mortality. Recently renamed metabolic-associated fatty liver disease (MAFLD), its prevalence is increasing, and the number of NAFLD patients worldwide is estimated to reach 56% in the adult population in the next 10 years [1]. NAFLD starts from simple steatosis and may progress toward non-alcoholic steatohepatitis (NASH), ultimately leading toward end-stage fibrotic liver disease, cirrhosis, and eventually hepatocellular carcinoma [2]. In the last few years, a growing amount of data is showing a strong link between irritable bowel syndrome (IBS) and NAFLD: in ultrasound studies, IBS patients were three times more likely to be affected by NAFLD compared to non-IBS patients (*p* < 0.001), and the proportion of NAFLD individuals with IBS increased with NAFLD severity (11% in mild, 28% in moderate, and 58% in severe NAFLD) [3]. The gut–liver axis refers to the bidirectional relationship between the gut and the liver and is influenced by the functioning of the mucosal epithelium and gut vascular barrier, which limit the systemic spread of microbes and toxins that cause or worsen a range of hepatic diseases. Furthermore, the gut-liver axis provides a direct conduit to the liver for the gut microbiota and their metabolic by-products (including secondary bile acids, ethanol, and trimethylamine). These gut–liver axis-related factors, including the host inflammatory response and integrity of the gut mucosal wall, likely contribute to the pathogenesis of MAFLD [4]. In particular, the accumulation of bile acids within hepatocytes can induce cytotoxicity by causing mitochondrial dysfunction and generating reactive oxygen species, which ultimately leads to apoptosis or necrosis [5]. Ethanol and its toxic metabolite, acetaldehyde, can activate the liver to produce inflammatory mediators, resulting in inflammatory liver injury; additionally, acetaldehyde can compromise intestinal barrier function by altering the expression of tight-junction proteins [6]. Trimethylamine N-oxide (TMAO) contributes to the progression of MAFLD through various mechanisms; inter alia, it promotes the expression of sterol regulatory element-binding protein-1c (SREBP-1c), a key regulator of liver lipid metabolism that enhances triglyceride synthesis, thus favoring the progression of hepatic steatosis [7]. Moreover, TMAO increases serum inflammatory factors that promote insulin resistance [8].

These observations have supported different research lines suggesting that the modulation of the microbiota and gut permeability could have a positive effect on liver steatosis and related conditions. In particular, a randomized, double-blind, placebo-controlled, phase 2a trial [9], in addition to highlighting the safety and efficacy of lubiprostone on reducing the levels of liver enzymes (transaminases and gamma-glutamyl transferase) in patients with NAFLD and constipation, represents the first proof-of-concept trial investigating intestinal permeability as a potentially novel therapeutic target in patients with NAFLD and constipation. Then, the obtained results suggest that manipulation of intestinal permeability might be a potential treatment approach for NAFLD with constipation.

On the other side, several studies report conflicting results on the effect of different prebiotic, probiotic, and symbiotic formulas on NAFLD and its laboratory markers [10,11]. In particular, recent reviews show high-quality preclinical studies and a few randomized controlled trials that highlight the effectiveness of probiotics, prebiotics, and symbiotics in NAFLD management. However, the effect of those formulas on NAFLD in clinical practice is still lacking, and the results of clinical studies are not consistent, for example, on parameters such as fatty liver index (FLI), liver transaminases, and triglycerides [10,11]. At the best of our knowledge, no clinical trial has been carried out until now on the effect of postbiotics (new preparations in the “biotic” field containing inanimate microorganisms and/or their metabolic products that confer a health benefit on the host.) on NAFLD.

In this context, the aim of our study was to clinically test the effect of butyrate supplementation on laboratory biomarkers of NAFLD.

## 2. Materials and Methods

This clinical trial was designed as a randomized, double-blind, placebo-controlled, parallel-group study, enrolling 50 Caucasian healthy individuals with NAFLD, defined as ultrasound-diagnosed liver steatosis and metabolic syndrome [12].

Exclusion criteria included any acute and chronic gastrointestinal disorder not controlled by stable treatment since at least 3 months, obesity (body mass index (BMI) > 30 kg/m^2^), type 1 and 2 diabetes, alcoholism, pregnancy and breastfeeding, and any medical or surgical condition potentially reducing the volunteers’ adhesion to the study protocol.

Enrolled individuals were adhering to a standard Mediterranean diet (defined as a plant-rich diet with extra-virgin olive oil as a main source of fats and a low content of salt and animal fats) for four weeks before randomization. The intervention period lasted 12 weeks. At each follow-up visit, individuals were evaluated for clinical status and the execution of the planned laboratory analyses.

The study fully adhered to the ethical guidelines outlined in the Declaration of Helsinki and the International Council for Harmonization of Technical Requirements for Registration of Pharmaceuticals for Human Use (ICH) Harmonized Tripartite Guidelines for Good Clinical Practice (GCP). The protocol received approval from the Ethical Committee of the University of Bologna, and all participants provided written informed consent.

### 2.1. Treatment

After a 4-week period of diet standardization, the enrolled individuals were randomized to receive either an indistinguishable placebo or active treatment tablets (kindly provided by Difass International, Cerasolo, Italy), two tablets per day, after dinner. The tested food for special medical purposes with functional release contained calcium butyrate (500 mg/tablet), zinc gluconate (zinc 5 mg/tablet), and vitamin D3 (500 IU/tablet). In calcium butyrate salt, the functional component is butyric acid, and the amount of calcium per se was negligible. The butyrate-based formula was tested in an organoid model derived from human intestinal cells to evaluate the modulation of the inflammatory process. Following the induction of inflammation, this formula showed significant anti-inflammatory activity and a greater effect on intestinal barrier integrity compared to a positive control and butyrate alone, as well [13], where the positive control was an in vitro-rebuilt intestinal epithelium exposed for 24 h to the stress agent LPS (liposaccarides from bacteria) in combination with the TNF-alpha cytokine. This formula showed significant anti-inflammatory activity (*p* < 0.05 for TNF-alpha and IL-6 levels, as % variation) and a greater effect on intestinal barrier integrity (*p* < 0.05 for TEER measures pre- and post-treatment, as % variation) compared to a positive control and butyrate alone.

At the randomization visit, the enrolled individuals received three boxes, each containing 60 tablets. The randomization process was centrally conducted using computer-generated codes. The randomization codes were kept in a sealed envelope, only opened after the study was completed and the data analyzed.

Throughout the study, individuals were instructed to take two tablets of the assigned treatment once daily, at approximately the same time each day, preferably in the evening. At the end of the trial, all unused tablets were collected for inventory, and participants’ compliance was assessed by counting the returned tablets.

### 2.2. Assessments

#### 2.2.1. Clinical Data and Anthropometric Measurements

The enrolled individuals were interviewed about their smoking habit, allergies, presence of gastrointestinal and other systemic diseases, and medications. Validated semi-quantitative questionnaires, including the food frequency questionnaire (FFQ), were used to assess dietary habits and recreational physical activity, as per usual practice in our research center [14,15]. Body weight, height, and waist circumference were measured by standardized methods. BMI was measured as body weight (kg)/square of height (m). Index of central obesity was measured as the ratio of waist circumference to height (normal if <0.5) [16].

#### 2.2.2. Laboratory Analyses

Biochemical analyses were conducted on venous blood collected after an overnight fasting of at least 12 h. Serum was obtained by adding disodium ethylenediaminetetraacetate (Na2EDTA) (1 mg/mL) and centrifuging the blood at 3000 rpm for 15 min at 25 °C. Following centrifugation, laboratory analyses were promptly carried out by trained personnel using standardized methods [17,18]. The following parameters were directly assessed: total cholesterol (TC), triglycerides (TG), high-density lipoprotein cholesterol (HDL-C), fasting plasma glucose (FPG), gamma-glutamyl transferase (gGT), glutamic-pyruvic (GPT) and glutamic-oxaloacetic transaminase (GOT), high-sensitivity C-reactive protein (hs-CRP), and creatine phosphokinase (CPK). LDL-C was obtained by the Friedewald formula. Visceral adiposity index (VAI) was calculated in males as waist circumference (WC)/[39.68 + (1.88 × BMI)] × (TG/1.03) × (1.31/HDL) and in women as WC/[36.58 + (1.89 × BMI)] × (TG/0.81) × (1.52/HDL), where TG and HDL-C values are included in mmol/L. The VAI normality cut-offs are 2.52 for individuals under 30 years, 2.23 for those aged between 30 and 42 years, 1.92 between 42 and 52 years, 1.93 between 52 and 66 years, and 2.00 for individuals over 66 years [19].

#### 2.2.3. Liver Steatosis Assessment

Liver steatosis was estimated with some validated indexes, such as hepatic steatosis index (HSI), lipid accumulation product (LAP), and fatty liver index (FLI), and then confirmed by standard ultrasound methods. HSI was calculated as 8·GPT/GOT ratio + BMI (+2 if woman; +2 if type 2 diabetes) [20]. LAP was calculated as [WC (cm) − 65]·[TG × 0.01143] for men and [WC (cm) − 58] [TG × 0.01143] for women [21]. FLI was calculated as [10^0.953^ × ln (TG) + 0.139 × BMI + 0.718 × ln (gGT) + 0.053 × WC − 15.745/(1 + 10^0.953^ × ln (TG) + 0.139 × BMI + 0.718 × ln (gGT) + 0.053 × WC − 15.745)] × 100 [22].

Liver steatosis was evaluated using a semi-quantitative method based on three qualitative criteria: parenchymal hyperechogenicity compared to kidney cortical echogenicity, posterior beam attenuation with standard settings, and blurred visualization of intrahepatic vessels and the diaphragm. The grading was defined as follows: grade 0 = no steatosis; grade 1 = mild steatosis, characterized by diffusely increased hepatic echogenicity but periportal and diaphragmatic echogenicity is still appreciable; grade 2 = moderate steatosis, diffusely increased hepatic echogenicity but periportal and diaphragmatic echogenicity is still appreciable; grade 3 = severe steatosis, diffusely increased hepatic echogenicity obscuring periportal as well as diaphragmatic echogenicity [23].

#### 2.2.4. Assessment of Tolerability

The study evaluated tolerability by continuously monitoring adverse events, clinical safety, laboratory results, and physical examinations. The subjective tolerability of the treatments was evaluated by a Visual Analogue Scale (1–10). The principal investigator designated a blinded, independent expert clinical event committee to categorize any adverse events occurring during the trial as not related, unlikely related, possibly related, probably related, or definitely related to the tested treatment [24].

### 2.3. Statistical Analysis

The sample size was determined based on the anticipated change in FLI. To detect a mean change in FLI of 5 units at 12 weeks with a power of 0.80 and an alpha error of 0.05, 22 individuals per group were needed. Accounting for a 5% dropout rate, the total sample size was 50 individuals (25 per group).

The normality of the variables was evaluated using the Kolmogorov–Smirnov test. Variables that were not normally distributed were log-transformed prior to further analysis. Baseline characteristics were compared using an independent Student’s *t*-test and the χ^2^ test, followed by Fisher’s exact test. Between-group differences were analyzed using repeated-measures ANOVA, with Tukey’s post hoc test for further evaluation. Means and standard deviations were used to represent all data. The analysis was conducted using the intention-to-treat approach with SPSS version 24.0 for Windows. All tests were two-sided, and a *p*-value of <0.05 was considered significant.

## 3. Results

**Figure 1:**
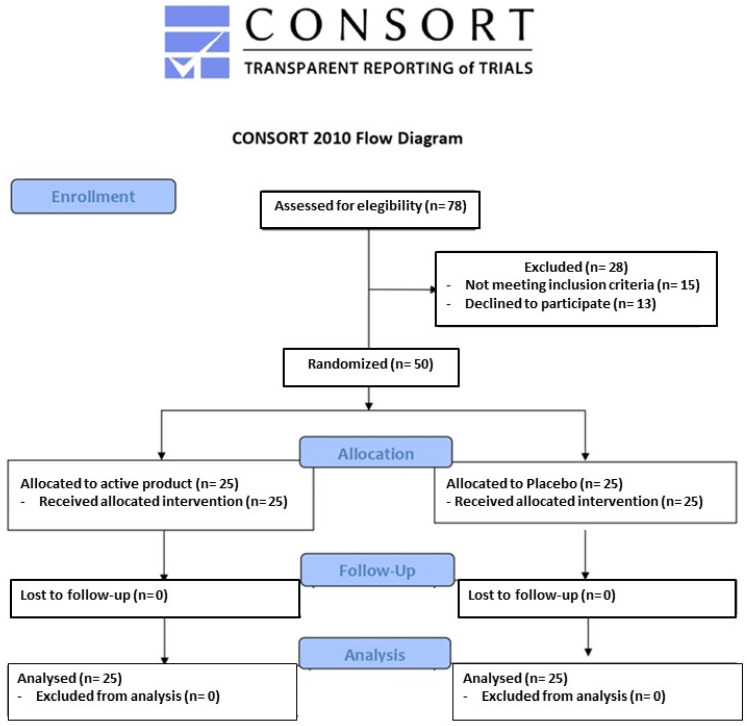
Individuals screened and enrolled in the trial.

Seventy-eight volunteers were screened, and 50 (27 men and 23 women) were randomized. All participants successfully completed the study as per its design. No enrolled subject experienced any subjective or laboratory adverse events, resulting in a dropout rate of 0%. Compliance with the treatment was approximately 100% in both active treatment and placebo treated groups (Figure 1).

Enrolled volunteers maintained similar dietary habits from the randomization until the end-of-study visit, without significant changes in total energy, salt, coffee, or alcohol intake.

No significant difference was detectable between the groups as regards the measured parameters at the screening (pre-run-in visit).

In the active treatment group, a significant decrease in TC and TG was observed at randomization visit vs. pre-run-in visit (*p* < 0.05) (Table 1).

**Figure 2:**
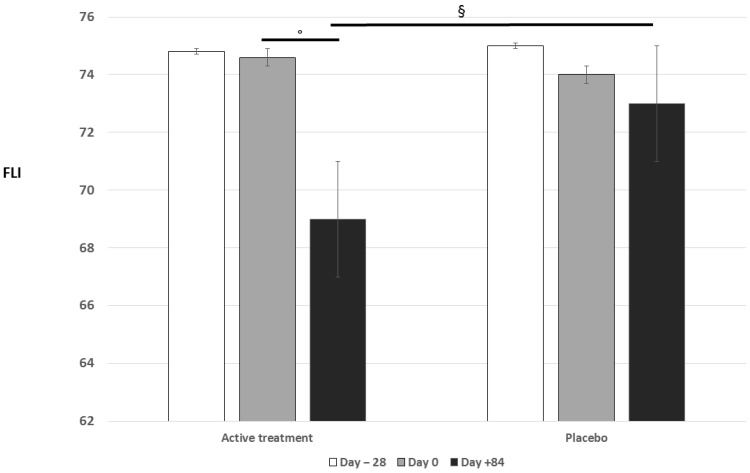
Changes in FLI after the active and placebo treatment. ° *p* < 0.05 vs. randomization visit; § *p* < 0.05 vs. placebo.

After active treatment, FLI improved significantly vs. baseline values (*p* < 0.05) and vs. placebo group (*p* < 0.05) (Figure 2). In particular, before active treatment, FLI was 74.6 ± 20.9, while after, it was 69.5 ± 14.7. In the placebo group, FLI remained unchanged (48.1 ± 8.2 vs. 45.5 ± 7.8). Moreover, after active treatment, TC, TG, gamma-gGT, and HSI improved significantly vs. baseline values (*p* < 0.05), and TC and TG improved vs. placebo group (*p* < 0.05), as well (Table 1).

In the placebo group, a significant decrease in TC and TG was observed at randomization visit vs. pre-run-in visit (*p* < 0.05). After treatment, TG further improved significantly, only compared to baseline values (*p* < 0.05) (Table 2).

## 4. Discussion

Despite a huge literature focusing on probiotics as metabolic modulators, other categories of “biotics” have recently attracted attention and scientific research focus, gradually shifting from viable probiotic bacteria towards non-viable paraprobiotics (non-viable inactivated microbial cells) and/or probiotic-derived biomolecules, so-called postbiotics [25]. The postbiotics are the complex mixture of metabolic products secreted by probiotics such as enzymes, vitamins, secreted proteins, short-chain fatty acids (SCFA), and other bioactive substances [26]. In 2019, the International Scientific Association for Probiotics and Prebiotics (ISAPP) convened a panel of experts specializing in various fields such as nutrition, microbial physiology, and gastroenterology, to review the definition of postbiotics. The panel defined a postbiotic as a “preparation of inanimate microorganisms and/or their components that confers a health benefit on the host” that must contain inactivated microbial cells or cell components, with or without metabolites that contribute to observed health benefits [24]. This definition would not include substantially purified metabolites in the absence of cellular biomass, in disagreement with what had been commonly accepted until then. The limitation of the definition of postbiotic to inactivated bacteria was contested by a panel of experts led by Aguilar-Toalá [27]. The debate is therefore still open. Probiotics techno-functional limitations, such as viability controls, have hampered their full potential applications in the food and pharmaceutical sectors. In particular, the main limitation of probiotics is notoriously the conservation of microbial vitality during the production processes and the conservation of finished products, which limits their effectiveness and diffusion in emerging countries, where it is difficult to guarantee the cold chain. Using postbiotics elicits several advantages, like their availability in their pure form, better availability of the production process for industrial scale-up, and ease of production and storage. Postbiotics may be well-suited for geographical regions where reliable cold chains are unavailable or where high ambient temperatures pose storage challenges for live microorganisms [24,25].

Examples of postbiotics include short-chain fatty acids (SCFAs) such as acetate, butyrate, and propionate [28]. Among SCFAs, butyrate has been extensively documented for its wide spectrum of positive effects and potential therapeutic use in human medicine [29]. Butyric acid is a key metabolite generated by the microbiome from the breakdown of non-digestible carbohydrates in the large intestine, serving as the main energy source for colonocytes. It also helps maintain the gut barrier, activates anti-inflammation signaling cascades, regulates histone acetylation, and impacts gene expression in various pathways, including the cell cycle, differentiation, and fatty acid metabolism [30]. In that way, butyrate enhances the intestinal barrier through specific mechanisms: positive regulation of the expression of claudin-1, ZO-1, and occludin in Cdx2-IEC and Caco-2 cells, resulting in increased transepithelial electrical resistance [31]; promotion of tight-junction assembly [32]; and enhancement of the mucous layer involved in the formation of the intestinal barrier [33].

NAFLD development and progression may be influenced by overnutrition, genetic predisposition, and changes in gut microbiota and intestinal barrier functions, potentially leading to increased bacterial endotoxin permeation. The composition of the gut microbiome is significantly impacted by diet. Diets rich in fat and sugar can change the diversity and functionality of the microbiome. For instance, high-fat diets can decrease the proportion of beneficial bacteria while increasing harmful ones. This imbalance may contribute to obesity and insulin resistance, which are risk factors for NAFLD. Obese individuals are known to have a higher Firmicutes/Bacteroidetes ratio compared to those of normal weight. This ratio is crucial for maintaining gut homeostasis [34]. The microbiome generates various metabolites, including SCFAs, that positively affect hepatic metabolism by modulating the immune response and enhancing insulin sensitivity. Conversely, an unhealthy diet can lead to the production of harmful metabolites, causing systemic inflammation and liver damage. Research in both preclinical and clinical models of NAFLD has revealed significant changes in the gut microbiome composition compared to healthy individuals, such as an increase in endotoxin-producing bacteria. These changes may contribute to liver inflammation and the progression of NAFLD to NASH. Additionally, patients with NAFLD often exhibit increased intestinal permeability, allowing bacteria and their endotoxins to enter the bloodstream and trigger an inflammatory response in the liver, exacerbating NAFLD. Consequently, dietary interventions, probiotics, prebiotics, and fecal microbiota transplants are being explored as potential therapeutic strategies to modulate the microbiome and enhance liver health in NAFLD/NASH patients. Recent scientific research emphasizes the critical role of the intestinal microbiome in the development of NAFLD and NASH, highlighting the influence of diet on the microbiome and liver health. Modulating the microbiome through dietary and therapeutic interventions is a promising area of research for treating NAFLD and NASH [35]. On the other hand, the progression of NAFLD is a complex process involving the accumulation of fats in the liver, often worsened by a high-fat diet and inflammation of adipose tissue. Recent research indicates that gut microbiome dysbiosis and impaired barrier function significantly contribute to this progression by allowing bacterial endotoxins to translocate and promote systemic inflammation [36]. A recent systematic review of preclinical studies highlighted the pleiotropic effects of butyrate in NAFLD, suggesting its potential as a nutritional aid in managing the disease. These benefits include modulation of intestinal homeostasis, reduction of intrahepatic triglyceride deposition, increased fatty acid oxidation, stimulation of beta-oxidation, and reduction of obesity [37].

In our double-blind, randomized clinical trial, we observed that 12-week dietary supplementation with a butyrate-based formula (calcium butyrate 500 mg/tablet) was able to significantly improve FLI vs. baseline and vs. placebo. Moreover, TC, TG, gGT, and HSI improved significantly vs. baseline values, and TC and TG improved vs. placebo group, as well. It is notable that FLI remains almost unchanged during the pre-run-in with a Mediterranean diet, unlike the lipid pattern, suggesting mechanisms different from nutritional ones in the regulation of liver function. Overall, this study suggests that supplementation with this butyrate-based formula is able to improve some NAFLD-related parameters in individuals affected by liver steatosis and metabolic syndrome.

To date, only preclinical studies have investigated the effects of postbiotics on NAFLD [38]. Specifically, in an NAFLD mouse model, butyrate alleviated hepatic steatosis by enhancing hepatic glucagon-like peptide-1 (GLP-1) sensitivity via increased GLP-1 receptor (GLP-1R) expression. Another NAFLD mouse model induced by a high-fat diet and treated with butyrate showed that butyrate acts as a negative regulator of liver lipogenesis, attenuates hepatic steatosis, and improves lipid profile and liver function [39]. However, the exact underlying mechanisms of butyrate remain unclear.

An animal model of ovariectomized mice provided evidence that estrogen deficiency worsens NAFLD, interfering with the gut microbiota, and butyrate supplementation can significantly attenuate it. Ovariectomized mice are a valuable tool for understanding estrogen deficiency, and this model provided that estrogen deficiency worsened NAFLD. The production of antimicrobial peptides (AMP) and the expression of intestinal epithelial tight junctions, including ZO-1 and occludin-5, were decreased in the ovariectomized mice compared to control mice. At the same time, an alteration in the composition of the gut microbiota and a decrease in the butyrate content are observed. These data suggest that alterations in gut microbiota and SCFAs due to estrogen reduction, along with disruptions in antimicrobial peptide (AMP) production and lipid metabolism, contribute to the development of non-alcoholic fatty liver disease (NAFLD). Therefore, SCFAs derived from the microbiota emerge as promising therapeutic targets for the clinical prevention and treatment of NAFLD [40]. These data are of particular interest, considering that estrogen has a known protective role against NAFLD, and fertile women are usually less likely to develop NALFD than men and post-menopausal women [41].

Although some studies have shown abnormal levels of SCFAs in NAFLD patients [42,43], to our knowledge, this is the first randomized controlled clinical trial investigating the effects of butyrate in NAFLD.

Of course, our study also has some limitations. First, the NAFLD diagnosis was not carried out with liver biopsy, which, however, was not indicated since we enrolled overall healthy individuals with non-significantly high levels of transaminases and/or gamma-GT. Since patients have these parameters within the norm, an improvement in the HSI cannot emerge, as expected. Furthermore, the study was relatively small, even if adequately powered for its aim. The duration of the study was short, so we cannot know whether prolonging the treatment could have induced further improvement in the recorded parameters. Finally, we have not tested if different dietary habits could have impacted the microbiota composition and the butyrate effect; however, the individuals enrolled in the trials followed a similar diet, which was further standardized before the enrolment and maintained constant during the trial.

In a context where NAFLD is a largely prevalent disease where evidence-based treatments are yet lacking, our preliminary investigation suggests for the first time that post-biotic supplementation could positively and safely impact some metabolic parameters related to this condition.

## 5. Conclusions

In this pilot randomized controlled study, we observed that the butyrate-based formula improved FLI and plasma lipid patterns in individuals affected by liver steatosis and metabolic syndrome. Further research is needed to confirm these suggestive findings, especially larger and longer-term studies involving individuals with a more severe degree of disease.

## Figures and Tables

**Table 1:** Trend of the measured parameters at screening, randomization and end of study visits in the active treatment group (*n* = 25).

Parameter	Pre-Run-In Visit (day −28)	Randomization Visit (day 0)	End of Study Visit (day 84)
Age (years)	61 ± 4		
Body mass index (kg/m^2^)	27.3 ± 2.1	27.1 ± 2.2	27.0 ± 1.9
Waist circumference (cm)	92 ± 5	90 ± 5	90 ± 6
Visceral adiposity index	2.9 ± 1.5	2.7 ± 1.1	2.6 ± 1.6
Steatosis severity	—	N. 17 Mild N. 8 Moderate	N. 2 Zero N. 17 Mild N. 6 Moderate
Fatty liver index	74.9 ± 17.7	74.6 ± 20.9	69.5 ± 14.7 °^§^
Hepatic steatosis index	47.5 ± 7.8	49.4 ± 8.8	43.1 ± 7.4 °
Lipid accumulation product	75.0 ± 37.2	69.8 ± 35.4	65.3 ± 33.9
Total cholesterol (mg/dL)	234 ± 12	228 ± 13 *	221 ± 11 °^§^
HDL-cholesterol (mg/dL)	46 ± 3	45 ± 4	46 ± 4
LDL-cholesterol (mg/dL)	137 ± 9	139 ± 9	134 ± 8
Triglycerides (mg/dL)	254 ± 23	221 ± 24 *	207 ± 23 °^§^
FPG (mg/dL)	98 ± 9	96 ± 10	96 ± 11
GOT (U/L)	23 ± 6	23 ± 5	22 ± 3
GPT (U/L)	25 ± 7	26 ± 7	23 ± 6
Gamma-GT (U/L)	31 ± 8	30 ± 9	26 ± 4 °
hs-C reactive protein (mg/L)	1.5 ± 0.3	1.4 ± 0.5	1.4 ± 0.4
Tolerability (VAS)	—	—	8 ± 1

* *p* < 0.05 vs. pre-run-in visit; ° *p* <0.05 vs. randomization visit; ^§^ *p* < 0.05 vs. placebo.

**Table 2:** Trend of the measured parameters at screening, randomization and end of study visits in the placebo group (*n* = 25).

Parameter	Pre-Run-In Visit (day −28)	Randomization Visit (day 0)	End of Study Visit (day 84)
Age (years)	60 ± 5		
Body mass index (kg/m^2^)	27.1 ± 2.2	27.0 ± 1.9	26.9 ± 2.0
Waist circumference (cm)	93 ± 6	91 ± 6	90 ± 7
Visceral adiposity index	2.9 ± 1.6	2.8 ± 1.3	2.6 ± 1.8
Steatosis severity	—	N. 16 Mild N. 9 Moderate	N. 1 Zero N. 16 Mild N. 8 Moderate
Fatty liver index	75.3 ± 16.9	74.1 ± 18.4	73.3 ± 15.6
Hepatic steatosis index	48.9 ± 7.1	48.1 ± 8.2	45.5 ± 7.8
Lipid accumulation product	74.7 ± 31.3	71.6 ± 30.7	68.8 ± 28.7
Total cholesterol (mg/dL)	237 ± 13	230 ± 11 *	228 ± 12
HDL-cholesterol (mg/dL)	45 ± 5	44 ± 5	45 ± 4
LDL-cholesterol (mg/dL)	142 ± 9	140 ± 10	139 ± 9
Triglycerides (mg/dL)	248 ± 19	229 ± 22 *	218 ± 23 °
FPG (mg/dL)	99 ± 10	97 ± 9	98 ± 10
GOT (U/L)	25 ± 5	24 ± 5	25 ± 6
GPT (U/L)	26 ± 6	25 ± 7	25 ± 6
Gamma-GT (U/L)	30 ± 9	28 ± 9	28 ± 7
hs-C reactive protein (mg/L)	1.3 ± 0.4	1.5 ± 0.4	1.4 ± 0.5
Tolerability (VAS)	—	—	8 ± 1

* *p* < 0.05 vs. pre-run-in visit; ° *p* < 0.05 vs. randomization visit.

## Data Availability

The original contributions presented in the study are included in the article, further inquiries can be directed to the corresponding author.
